# Hemostatic materials in wound care

**DOI:** 10.1093/burnst/tkab019

**Published:** 2021-09-15

**Authors:** Peiyu Yu, Wen Zhong

**Affiliations:** Department of Biosystems Engineering, University of Manitoba, 75A Chancellor's Circle, Winnipeg, MB, R3T 2N2 Canada; Department of Biosystems Engineering, University of Manitoba, 75A Chancellor's Circle, Winnipeg, MB, R3T 2N2 Canada

**Keywords:** Hemostasis, Hemorrhage, Hemostatic materials, Wound healing, Conventional hemostatic materials, High-performance hemostatic materials

## Abstract

Blood plays an essential role in the human body. Hemorrhage is a critical cause of both military and civilian casualties. The human body has its own hemostatic mechanism that involves complex processes and has limited capacity. However, in emergency situations such as battlefields and hospitals, when the hemostatic mechanism of the human body itself cannot stop bleeding effectively, hemostatic materials are needed for saving lives. In this review, the hemostatic mechanisms and performance of the most commonly used hemostatic materials, (including fibrin, collagen, zeolite, gelatin, alginate, chitosan, cellulose and cyanoacrylate) and the commercial wound dressings based on these materials, will be discussed. These materials may have limitations, such as poor tissue adhesion, risk of infection and exothermic reactions, that may lessen their hemostatic efficacy and cause secondary injuries. High-performance hemostatic materials, therefore, have been designed and developed to improve hemostatic efficiency in clinical use. In this review, hemostatic materials with advanced performances, such as antibacterial capacity, superhydrophobicity/superhydrophilicity, superelasticity, high porosity and/or biomimicry, will be introduced. Future prospects of hemostatic materials will also be discussed in this review.

Highlights

- Hemostatic mechanism of the human body.
- Drawbacks of conventional hemostatic materials.
- High-performance hemostatic materials that promote wound healing.

## Background

Blood is composed of erythrocytes, leukocytes, platelets and plasma, making up about 7–8% of total body weight. Blood in the human body is involved in several essential processes, including transporting oxygen and other nutrients to different organs, preventing excessive blood loss and regulating body temperature [1]. However, in battlefields, hospitals and other emergency situations, uncontrolled hemorrhage causes over 30% of traumatic deaths, half of which happen at the prehospital stage. It is also suggested that 50% of military mortality is caused by bleeding [2, 3]. Excessive bleeding can cause severe damage, including hemorrhagic shock, hypothermia, hypotension, multiple organ failure, acidosis and infections [4, 5]. Therefore, hemostasis becomes an important step for trauma treatment.

**Figure 1.**
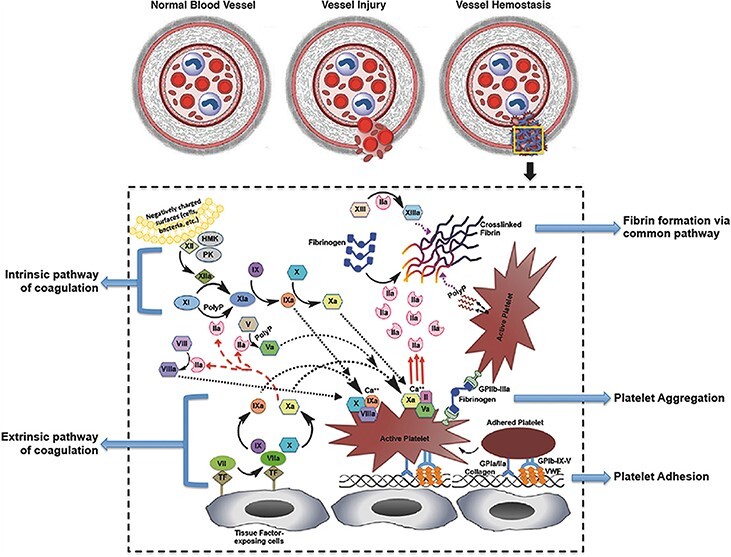
Schemes of the intrinsic hemostatic mechanisms of the human body [5] (Copyright 2017 by John Wiley & Sons, Inc., New Jersey, USA)

The intrinsic hemostatic mechanism of the human body has a limited capacity and may need assistance via hemostatic materials or devices for rapid hemostasis, particularly in emergency situations [6]. In clinical practice, compression with cotton gauze and wound closure with sutures or staples are the most frequently used methods to stop bleeding. Recently, a variety of hemostatic materials have been generated for the industry, namely, collagen [7], zeolite [8], gelatin [9], alginate [10], chitosan [11], cellulose [12] and cyanoacrylate [13]. However, the hemostatic efficiency of these materials cannot fully meet clinical requirements [6, 14, 15]. Therefore, considerable efforts have been made in recent years to improve high-performance hemostatic materials.

A desirable hemostatic material should generally have rapid and sustainable hemostatic efficacy, biocompatibility, biodegradability, non-cytotoxicity and firm adhesion in a moist environment. Furthermore, ease of use, shelf life and cost are also major factors to be considered in the design and engineering of hemostatic materials. [6, 15].

In this review, a description of the progress of hemostatic materials is given, starting with an introduction to the intrinsic hemostatic mechanism of the human body and existing hemostatic methods, followed by a discussion on hemostatic materials that have been used clinically. High-performance hemostatic materials, including those that are antibacterial and biomimetic, will be described. The future outlook of high-performance hemostatic materials will be briefed by the end of this review.

## Review

### Hemostatic mechanisms and current hemostatic methods

Hemostasis is a complicated process that converts an unstable platelet plug into stable fibrin and includes two steps, primary hemostasis and secondary hemostasis (the coagulation cascade) (Figure 1). In the primary hemostasis stage, vessels contract to diminish blood loss from the wound and procoagulant proteins and factors are secreted. Meanwhile, activated platelets form an initial platelet plug in the injured vascular wall. Other platelets are also activated and aggregated in the blood to form a hemostatic plug to avoid hemorrhage. The secondary hemostasis stage (coagulation cascade) is the process of forming fibrin clotting at the site of initial hemostatic plug, including intrinsic pathway, extrinsic pathway and common pathway. In the intrinsic pathway, coagulation factor X is activated in the presence of Ca^2+^ and platelet-secreted phospholipid membrane. In the extrinsic pathway, in the presence of Ca^2+^, tissue factor can combine with active coagulation factor VII to form a factor VII–tissue factor complex. In the common pathway, activated factor X can synthesize fibrin with the participation of Ca^2+^, platelet-secreted phospholipid membrane and activated factor XIII. The fibrin is used to bolster the platelet plug that is formed in the primary hemostasis stage [5, 6].

Various hemostatic methods have been used to stop bleeding in different situations. For example, Ferreiral *et al.* used nylon cable ties to prevent hemorrhage for castration of male cattle and found that the nylon cable ties is an effective and economic hemostatic material [16]. Itoi *et al.* used an endoscopic hemoclip to treat uncontrolled sphincterotomy bleeding [17]. Cho *et al.* used sutures for uterine hemostasis in cesarean delivery to prevent uncontrolled postpartum bleeding to avoid hysterectomy [18]. Maeda *et al.* illustrated that the stapler can completely stop bleeding for mesenteric vessels in surgery for a prolapsed transverse colostomy compared with a hand-sewn technique [19]. Other common hemostatic methods can be found in other review papers [20, 21]. However, surgical procedures such as sutures and staples may not be suitable for all types of wounds, especially wounds with significant tissue loss, necrosis, uneven edges or infections [22]. In such situations, hemostatic materials or wound dressings are more effective in controlling hemorrhage and assisting wound healing.

### Conventional hemostatic materials

**Figure 2.**
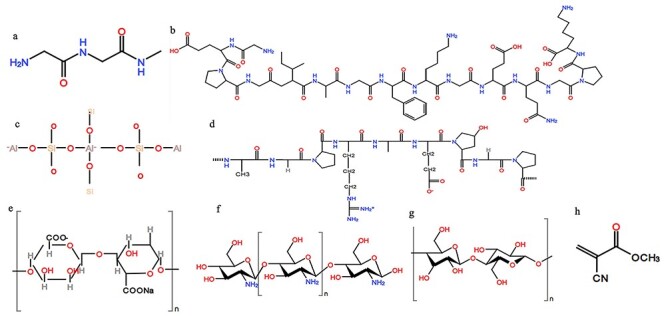
The chemical structures of fibrin **(a)**, collagen **(b)**, zeolite **(c)**, gelatin **(d)**, sodium alginate **(e)**, chitosan **(f)**, cellulose **(g)** and cyanoacrylate **(h)**

When hemorrhage is severe and beyond the capacity of the intrinsic hemostasis mechanism of the human body, hemostatic materials are needed to stop bleeding. The mechanism of hemostatic materials usually involves 2 pathways, namely the active pathway and the passive pathway. The active pathway works to trigger hemostasis by specifically initiating the coagulation cascade, while the passive pathway achieves hemostasis via the specific surface properties of the hemostatic materials, such as hemocompatibility and anti-infection. In the hemostatic process, metal ions, particularly Ca^2+^, play an important role because Ca^2+^ participates in several essential steps in the coagulation cascade [6]. Conventional hemostatic materials are introduced in this section. Table 1 shows the commercial hemostatic materials in the market. Figure 2 shows the chemical structure of conventional hemostatic materials.

**Table 1:** Examples of commercial hemostatic materials

**Materials**	**Brand name**	**Manufacturer**	**Pros (+) and cons (−)**
Fibrin sealant	Evicel®	Omrix	+: easy to use; effective hemostatic performance with heparin −: may cause blood-borne disease [23]
	Tisseel®	Baxter Healthcare	
	Crosseal®	Omrix	+: shorter hemostasis time; fewer postoperative complications; less blood loss [24] −: neurotoxicity [23]
	Quixil®	Omrix	
Oxidized cellulose	Surgicel Original®	Johnson & Johnson	+: antibacterial ability; easy to use and handle −: lower pH causes inflammation and hemolysis [23, 25]
	Surgicel Nu-Knit®		
	Surgicel Fibrillar®		
	Interceed®		
	Gelitacel®	Gelita Medical	
Gelatin	Surgifoam®	Johnson & Johnson	+: fewer complications; absorbed within 4–6 weeks; neutral pH −: high swelling ability; foreign body reaction [23, 25]
	Gelfoam®	Pfizer	
	Gelfilm®		
	Geli putty®	Gelita Medical	
	Gelita-spon®		
Collagen	Instat®	Johnson & Johnson	+: reducing blood loss; large surface area; stops bleeding within 2–5 minutes −: less efficacy for patients with thrombocytopenia or coagulopathies; may cause neural pain or numbness [25]
	Helitene®	Integra	
	Helistat®		
	Avitene®	Davol	
	Avitene flour®		
	Avitene Ultrafoam®		
	Endo Avitene®		
	Avitene Ultrawrap®		
	Surgiflo®	Johnson & Johnson	
Cyanoacrylate adhesives	Dermabond®	Johnson & Johnson	+: rapidly stops bleeding −: cytotoxicity
	Omnex®	Ethicon	
Polyethylene glycol	CoSeal®	Baxter Healthcare	+: degrades within 4 weeks; directly applied to the tissue surfaces −: less swelling ability (up to 4 times of original volume) [25]
Zeolite	QuikClot®	Z-Medica	+: decreases blood loss −: exothermic reaction [8]
Chitosan	Celox®	MedTrade Products Ltd	+: reduced compression time (1 minute) [26] −: cannot be used for a long time [27]
	HemCon® bandage	HemCon Medical Technologies Inc., Portland, OR	+: antibacterial property; useful on severe arterial hemorrhage [28] −: more expensive than Celox; longer treatment time (5 minutes) [28, 29]

**Blood-derived hemostatic materials: fibrin, thrombin and fibrinogen** Fibrin is one of the main components in the hemostatic clot formation and can be derived from human plasma [5, 15]. In 1984, Rousou *et al.* proved that fibrin glue is a simple, effective and low-cost hemostatic agent for unsutured surgical bleeding [30]. In 1990, Raccuia *et al.* measured the hemostatic efficiency of oxidized cellulose, collagen and fibrin glue in a rat kidney injury model and found that the fibrin glue has superior hemostatic ability compared to the other 2 materials [31]. Delgado *et al.* reported that, in a porcine grade V liver injury model, a fibrin patch effectively decreased blood loss and increased the survival rate [32]. Krishnan *et al.* indicated that fibrin-based sheets can stop bleeding rapidly (about 3–5 seconds in a rabbit ear artery model and less than 3 minutes in a rat liver model) and are degraded within 15 days in rats [33]. As the major components of fibrin, thrombin and fibrinogen have been developed as hemostatic materials. [34]. For example, Li *et al.* prepared a thrombin/graphene sponge that can block bleeding within 100 seconds in a rat tail injury model, which is much faster than crosslinked graphene sponges (200 seconds) and gauze with thrombin (250 seconds). After 6 months of storage, it can block hemorrhage within 118 seconds [35]. The immune response in pigs to the thrombin/fibrinogen wound dressings was also investigated. The results demonstrated that, within 6 months, the immune response of swine was normal. Hence, thrombin and fibrinogen have been approved as safe in animals as hemostatic materials [36].

**Collagen** Collagen is the most abundant protein in a mammal’s body, constituting the extracellular matrix of most connective tissues [5, 37]. Collagen-based hemostatic materials can activate the intrinsic pathway of the secondary hemostatic process [25]. The first commercial collagen-based hemostatic material was produced in the 1970s [25]. In 1974, Morgenstern reported the use of microcrystalline collagen hemostat (Avitene®) to control splenic bleeding in a dog. The result showed that the material can stop bleeding within 5 minutes without any side effect and is degraded within 6 weeks [38]. Cheng *et al.* extracted collagen from jellyfish to prepare a collagen sponge for hemostasis. The results indicated that the non-cytotoxic collagen sponge can stop bleeding within 5 minutes, which is 10 minutes less than the medical gauze that was used as a control [7].

**Zeolite and kaolin** Zeolite and kaolin, which are microporous aluminosilicate minerals with large surface areas, have shown high hygroscopicity and excellent hemostatic performance. The hemostatic mechanism of zeolite is via the absorption of blood and the release of Ca^2+^ into the blood and to spur the intrinsic path of coagulation cascade [5, 39]. An example of a commercial zeolite-based hemostatic material is QuikClot®, which has been proved to have good hemostatic efficacy in different animal models, including a swine groin injury model, a porcine grade V liver model and a lethal rabbit groin injury model [5, 40–43]. Laurenti *et al.* explored the hemostasis of zeolite-based procoagulant hemostatic agents, namely micro- and nanometric faujasite zeolites, and indicated that calcium ions exchanged nanometric faujasite zeolites (Nano-FAU/Ca) can enhance hemostatic performance significantly [44].

Kaolin powder has also been used in hemostatic dressings. A sponge impregnated with kaolin and graphene was developed and shown to be non-cytotoxic and biocompatible; it also blocked bleeding within 73 seconds in a rabbit injury model [39]. Sun *et al.* prepared a microsphere containing chitosan and kaolin and demonstrated that kaolin can improve the efficiency of hemostasis. The result shows that the time to hemostasis for composite microspheres (120 seconds and 99 seconds) was shorter than that of chitosan microspheres (183 seconds and 134 seconds) in rat tail and liver models. Meanwhile, the chitosan/kaolin microspheres have lower blood loss than the chitosan microspheres in the rat model [45].

**Gelatin** Gelatin is a water-soluble protein is derived from collagen hydrolysis. Gelatin is highly absorbent and can absorb 5–10 times its dry weight in water [46, 47]. Gelatin and microbial transglutaminase were used to prepare an *in situ* gel-forming adhesive that can form gels within 30 minutes under damp conditions and stop bleeding in 2.5 minutes in a rat liver and femoral artery injury model and 4 minutes in a porcine model [48]. A novel chemical crosslinked gelatin sponge was prepared and used in a 12-year-old male patient who was suffering from bleeding of a pharyngeal angiofibroma. The result showed that the gelatin sponge can stop bleeding immediately and degrades after 2 weeks [9].

**Alginate** Alginate, a natural polymer with negative ions, can be extracted from seaweed. Because of its biocompatibility and low cytotoxicity it is commonly used for medicinal purposes, including in wound dressings. Alginate can form a gel or be crosslinked with divalent ions, such as Ca^2+^. Alginate dressings are used to treat exuding wounds and may accelerate wound healing by creating a damp wound healing environment. It is also easy to remove the alginate dressing from a wound without causing additional injury [6, 10, 49]. Thomas *et al.* reported that alginate wound dressings can activate human macrophages to promote wound healing [10].

**Chitosan** Chitosan is a natural cationic polysaccharide that is made from deacetylated chitin and widely applied in different fields, such as the food and cosmetic industries [11, 50]. Because of its biocompatibility, biodegradability, non-cytotoxicity and antibacterial properties, chitosan can be used in tissue engineering [6]. Although the application of chitosan as a hemostatic material can be traced back to the early 1980s, the hemostatic mechanism of chitosan is still not well understood [50, 51]. Janvikul *et al.* explored the *in vitro* hemostatic efficacy of chitin, chitosan and their derivatives. Their results showed that a chitosan derivative, N,O-carboxymethylchitosan, can accelerate the hemostasis process *in vitro* and activate platelets most effectively [52]. Chitosan has also been used in combination with other chemicals and materials in developing hemostatic materials. For example, a chitosan-based wound dressing loaded with inorganic additives (aluminum chloride, iron (III) sulfate and aluminum sulfate) and levofloxacin was fabricated. In this system, inorganic additives can stop hemorrhage and levofloxacin can be released to provide antibacterial functions. The results showed that the chitosan-based materials with aluminum sulfate and levofloxacin had the highest blood absorption capacity and augmented the hemostatic capacity in an *in vivo* mice injury model [53]. Maevskaia *et al.* prepared a chitosan-based wound dressing incorporated with chitin nanofibrils. Compared with 2 commercial hemostatic products (Surgicel and TachoComb), the chitosan sponges with 0.5% chitin nanofibrils demonstrated faster hemostatic ability in both rat femoral and vein artery injury models [54].

**Cellulose-based materials** Cellulose is a linear biopolymer derived from delignified wood fibers [55]. Recently, cellulose, especially nanocellulose and its derivatives, has gained widespread attention in the biomedical field because of its biocompatibility, negative surface charge, high surface area, non-toxicity and low cost [6, 56]. Oxidized cellulose is a popular clinical hemostatic material that was first used in 1942. The first hemostatic product based on regenerated oxidized cellulose, Surgicel®, appeared in 1960 [25]. However, compared to oxidized regenerated cellulose, oxidized non-regenerated cellulose showed better hemostatic efficacy due to its fiber structures, which are frayed and therefore provide a larger surface area [57].

**Cyanoacrylate** Cyanoacrylate is a synthetic hemostatic polymer with good tissue adhesive properties that has been used as a hemostatic material since 1942 [5, 25]. Cyanoacrylate has been commonly used as a clinical tissue adhesive due to its rapid hemostasis, reducing keloid formation, decreasing pain scores and low cost [13, 58]. In recent years, the cyanoacrylate derivatives 2-butyl cyanoacrylate and 2-octyl cyanoacrylate have gained attention because they can improve the strength and flexibility of cyanoacrylate-based materials. Jiang *et al.* prepared a self-assembling 2-octyl cyanoacrylate film that can endure 147 mmHg of pressure and exhibits a rapid hemostatic ability (within 1 minutes) in a pig liver model [13].

Although the materials mentioned above have good hemostatic performance, their shortcomings are also evident. For example, fibrin is extracted from the blood of pooled donors and may therefore pose a risk of viral infection. Nanofiltration can reduce the risk of viruses (such as human immunodeficiency virus, hepatitis B virus, hepatitis C virus and hepatitis A virus) but it may still be difficult to eliminate them [15, 59]. Swelling of collagen limits its usage in infected areas because it is likely to cause injuries in adjacent tissues and structures [60]. Zeolite can absorb water and has exothermic reactions which can cause wound burns and inflammation [2, 61]. There is a report on a modified QuikClot that reduces the heat release. However, the temperature in the wound is still higher (40.3°C) than human body temperature (37°C) [8]. Cyanoacrylate-based hemostatic agents have been reported to be toxic and cause infection and tissue necrosis [15, 58]. Therefore, there has been an emergent need to develop high-performance hemostatic materials to satisfy the requirements of clinical applications.

### High-performance hemostatic materials

The hemostatic process is complicated. Although the human body has its own hemostatic mechanism, it may not be sufficient for massive bleeding. Various methods, including cautery, sutures and lasers, have been developed to stop bleeding in surgery and the battlefield; however, not all of them are effective in all situations [59]. Hemostatic agents have been used to improve hemostatic efficiency and decrease the hemostasis time. Previous research illustrated that hemostatic agents can minimize blood loss and reduce the risk of surgical complications [62, 63]. High-performance hemostatic materials that promote hemostasis and wound healing will be discussed in this section.

**Antibacterial hemostatic agents** Conventional hemostatic materials, such as medical gauze and fibrin, can transmit diseases and cause infections in hospitals and military camps or under emergency situations, especially when a sterile environment is not available for trauma patients [64, 65]. Antibiotics are used clinically to treat bacterial infections; however, overuse of antibiotics may lead to drug resistance problems [12]. To minimize the usage of antibiotics, antibacterial agents have been used to endow hemostatic materials with antibacterial properties. Antibacterial agents include organic (i.e. quaternary ammonium salts) and inorganic agents (i.e. silver ions [65, 66] and graphene oxide [67]) [12, 68].

**Figure 3.**
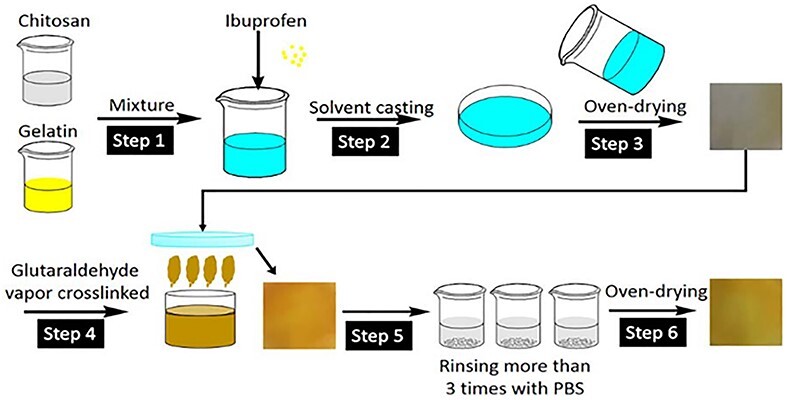
Scheme of preparing ibuprofen-loaded chitosan/gelatin composite films [70]. (Copyright 2017 by John Wiley & Sons, Inc., New Jersey, USA)

Chitosan has an intrinsic antibacterial efficacy which can be further enhanced by loading antibacterial agents, such as silver sulfadiazine [69]. Li *et al.* formulated chitosan/gelatin composite membranes loaded with ibuprofen (Figure 3). In the antibacterial experiments against *Staphylococcus aureus* (*S. aureus*) and *Escherichia coli* (*E. coli*), the composite films displayed an excellent antimicrobial effect, especially against *S. aureus*, and in a rabbit liver injury model the ibuprofen-loaded chitosan/gelatin films displayed excellent hemostatic performance [70].

**Figure 4.**
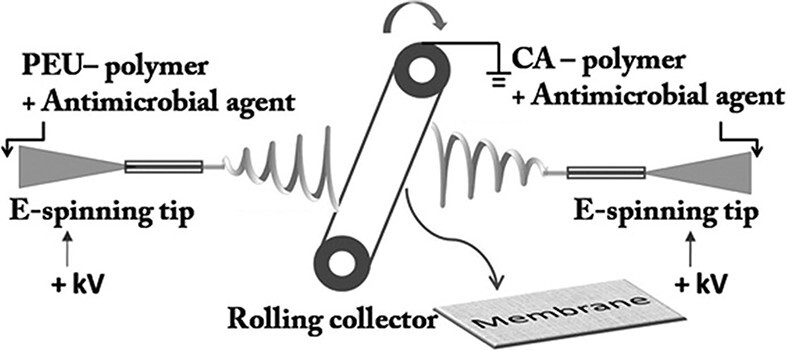
The process of preparing electrospun fibers. *PEU* polyester urethane, CA cellulose acetate, *E-spinning tip* electrospinning tip. [12] (Copyright 2012 by John Wiley & Sons, Inc., New Jersey, USA)

**Figure 5.**
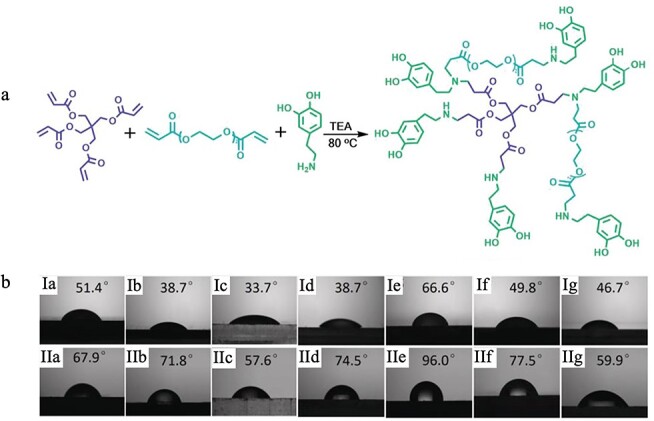
A hyperbranched polymer (HBP) with hydrophilicity. (**a**) Michael addition reaction process of HBP adhesives; (**b**) Contact angles of HBP adhesives (I) and water (II) on ceramic (Ia,IIa), iron sheet (Ib,IIb), PMMA (Ic,IIc), PET (Id,IId), PTFE (Ie,IIe), PE (If,IIf), and glass (Ig,IIg). *PMMA* poly(methyl methacrylate), *PTFE* poly(tetrafluoroethylene), *PE* polyethylene *HBP* hyperbranched polymer. [95] (Copyright 2019 by John Wiley & Sons, Inc., New Jersey, USA)

**Figure 6.**
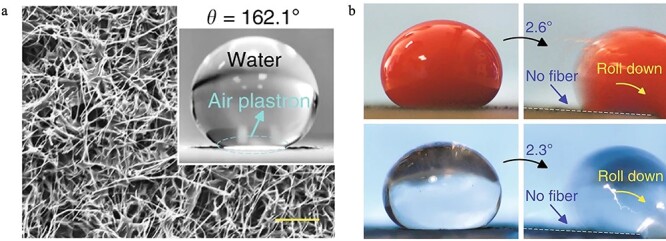
Superhydrophobic property of carbon nanofibers (CNFs). (**a**) Scanning electron microscopy image of the superhydrophobic CNF/PTFE Ti surface and water contact angle of the surface. (**b**) Blood and platelet-poor plasma droplets with anti-thrombin rolled down rapidly on the CNF/PTFE Ti surface with a small tilt angle [96]. PTFE poly(tetrafluoroethylene)

**Figure 7.**
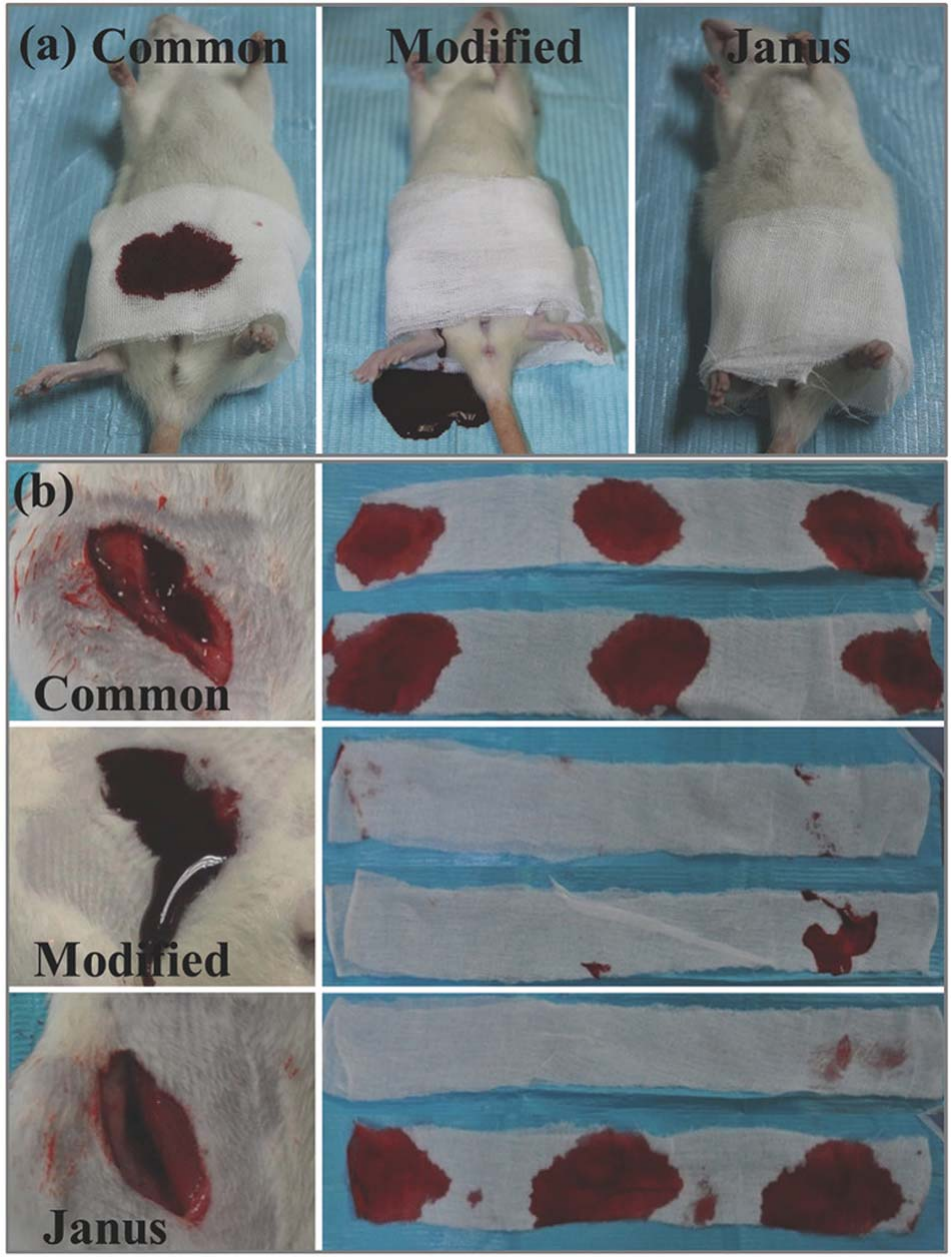
In vivo hemostatic assay of Janus fabric. (**a**) Hemostatic performance in the rat model: The rat with injured femoral artery wrapped with bilayer common gauze, bilayer modified gauze, and Janus gauze, respectively; (**b**) The wounds after removing the gauze and the used gauze. [94] (Copyright 2018 by JohnWiley & Sons, Inc., New Jersey, USA)

**Figure 8.**
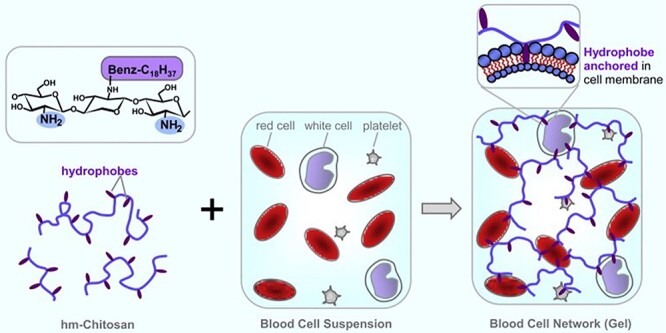
Blood gelation mechanism of hm-chitosan. [97] (Copyright 2018 by Elsevier Ltd, Amsterdam, Netherlands)

Metal ions, including silver (Ag^+^), copper (Cu^2+^) and zinc ions (Zn^2+^), also display antimicrobial properties because the positively charged metal ions can combine with the negatively charged bacterial membranes to interrupt normal bacterial functions and crush the structures, leading to cell death and achieving their antibacterial aims [71–77]. Hu *et al.* prepared a wound dressing containing nanoporous bioglass with silver [78] that had a high surface area and water absorption rate. The hemostatic dressing exhibited a great antibacterial ratio (99% in 12 hours) against *E. coli*. A rabbit injury model showed that the hemostatic dressing has an outstanding hemostatic performance and can reduce the hemorrhage time. Pourshahrestani *et al.* proved that gallium ions have antibacterial abilities and can accelerate the hemostatic process [79].

Although inorganic antibacterial agents are more stable and have a longer shelf life than organic agents, inorganic nanoparticles can damage the human cardiovascular system [12, 68]. Therefore, organic antibacterial agents are also extensively used in studies. Polyhexamethylene biguanide (PHMB) is a polymeric antibacterial agent that has been used to disinfect swimming pools [12]. PHMB was also integrated into electrospun nanofibers (Figure 4) consisting of cellulose acetate and polyester urethane to fabricate hemostatic nanofibrous films. *In vitro* antibacterial experiments showed that the films containing PHMB had a bacterial reduction rate of over 96% against *E. coli*. The diffusion speed of PHMB can be controlled in a sustained rate; hence, the films provide a long-term antibacterial property. An *in vivo* rat skin wound model indicated that the nanofibrous membranes have a good wound healing performance. Furthermore, poly(N,N-dimethylamino-2-ethyl methacrylate) (PDMAEMA) has been proved to have hemostatic and antimicrobial properties [80]. In another study, poly(D- or L-)lactide with PDMAEMA was used to fabricate stereocomplex-based hemostatic materials [81]. An *in vitro* blood adhesion experiment showed that mats containing PDMAEMA can absorb and adhere human blood. However, PLA-b-PDMAEMA mats can adhere a smaller number of *S. aureus* and *E. coli* cells.

Previous studies proved that oxidized regenerated cellulose (ORC) with metal ions showed great antimicrobial properties [82]. An ORC gauze treated with chitosan and NaOH/C_2_H_5_OH was reported [83]. *In vivo* hemostatic experiments showed the minimum and maximum hemostasis times of the gauze were 145 seconds and 325 seconds in a rabbit liver injury model, respectively, and 155 seconds and 320 seconds in rabbit ear artery injury model, respectively. The antibacterial experiments displayed that the antimicrobial efficiency against *S. aureus* and *E. coli* reached 99.9% for the ORC gauze.

**Superhydrophobic or superhydrophilic hemostatic materials** Based on surface properties, materials can be categorized as hydrophobic or hydrophilic, which can be differentiated by their water contact angles. Water contact angles of hydrophobic surfaces are larger than 90°. When the angle is higher than 150°, the material is regarded as superhydrophobic. In contrast, a surface with a water contact angle smaller than 90° is hydrophilic, and if it is below 10°, it is superhydrophilic [84, 85].

Superhydrophobic and superhydrophilic surfaces are common in nature and can be achieved by biomimetic design. Superhydrophobic surfaces, for example, may be inspired by duck feathers or lotus leaves, which are natural superhydrophobic materials [86, 87]. It has been found that the nanostructure of lotus leaves contributes to the high water contact angles on their surface [88]. On the other hand, superhydrophilicity was initially discovered in human tears because they can spread and form a membrane to prevent any damage to the eyes; fish scales provided a new inspiration for superhydrophilic surfaces [86, 89]. Generally, superhydrophobic or superhydrophilic materials can be obtained by manipulating the roughness and microstructure of their surfaces [84, 90] and have been applied in water collection, printing, self-cleaning, sensors, bio-adhesion, anti-fogging, liquid–liquid separation, liquid transport, anti-fouling and water/oil separation [86, 91, 92].

The properties of superhydrophobicity and superhydrophilicity can also be used in hemostatic processes. Superhydrophobic surfaces may attract proteins and form a film on the wound to prevent further loss of blood [93, 94]. Hydrophilic materials, on the other hand, can extract water from the blood to speed up the coagulation process [94]. Normally, the superhydrophobic material can be coated on the outside of the hydrophilic wound dressing to prevent blood loss. For instance, Cui *et al.* designed a hyperbranched polymer (HBP) adhesive with a hydrophobic backbone and a hydrophilic adhesive side chain (Figure 5) [95]. When the HBP comes in contact with liquid (such blood or water), the hydrophobic backbone chains can self-aggregate rapidly and the hydrophilic groups can be exposed to water and adhere to different material surfaces under moist environments. The touch angles of HBP adhesives were all lesser than 90° (minimum 33.7° and maximum 51.4°) (Figure 5). *In vivo* hemostatic experiments showed that the HBP adhesives have good hemostatic performance and can stop bleeding within 1.5 minutes in a rat femoral artery injury model and seal the wound within 4 seconds in a pig liver model.

Li *et al.* synthesized a superhydrophobic hemostatic dressing by immobilizing carbon nanofibers (CNFs) [96].The water contact angles of two surfaces, CNFs/polytetrafluoroethylene Ti surface and CNFs/polydimethylsiloxane Ti surface, are 162.1° and 154.9°, respectively (Figure 6). The superhydrophobic property of CNFs may alleviate blood loss and increase the bacteria reduction rate. In a rat injury model, compared to cotton gauze, the CNF gauze could control bleeding in 3 minutes and due to its superhydrophobic property, the CNF gauze is easy to peel without any wound tearing or hemorrhage.

Cotton gauze and paraffin were used to prepare a Janus fabric with superhydrophobic and superhydrophilic properties [94]. Cotton gauze has an inherent hydrophilic property, with one side coated with paraffin to endow hydrophobic properties. Therefore, the 2 sides of the Janus fabric have different surface properties of superhydrophobicity and superhydrophilicity, respectively. The water contact angles for the 2 sides are 154° and 0°, respectively. In rat injury models, compared with control groups, the Janus fabrics can reduce blood loss (an average decrease of 64%) (Figure 7) and prolong the survival time of rats (increased by 41%).

Dowling *et al.* introduced a self-assembled amphiphilic biopolymer that was prepared by using a hydrophobically modified chitosan (hm-chitosan) [97]. Upon contact with human blood, the polymer changed from a liquid state to a gel (Figure 8); the reversal of the gelation was achieved by adding α-cyclodextrin because the hydrophobic polymers can be released from blood cells and inserted into cyclodextrin, and the internal structure of the gel was destroyed. In a rat femoral artery model, the material can reduce the hemostasis time by 90% compared with the control group. The hm-chitosan was attached to the wound in the pig femoral artery model and the wound was successfully clotted when the material was removed after 3 hours. Therefore, the potential for hm-chitosan to be used as a low-cost wound dressing with high hemostatic efficiency is encouraging.

**Biomimetic hemostatic materials** Biomimetic materials research has a long history and is developing rapidly. Biomimetic materials are inspired by nature and examples include butterfly wings, bones, spider silks and mussels [98, 99]. To design a biomimetic material, the structure and/or physical/chemical nature of the natural material are explored and imitated to duplicate the special function of the material [98]. Recently, biomimetic materials have been applied in various fields, such as tissue engineering [100, 101], myocardial tissue [102], actuator materials [103], drug delivery [104] and conductive film [105].

Most hemostatic adhesives may lose their efficiency underwater or in a wet environment because water molecules can impair the inter-surface physical adhesive forces and may change chemical bonds [106]. Wound dressings with high hemostatic efficiency in the wet medium should be developed to meet such demands. Some marine organisms, such as mussels, have been found to have a natural ability to attach to different surfaces under the sea to gain necessary resources, avoid predators and improve genetic levels [107]. Therefore, mussel-inspired hemostatic materials have been fabricated.

**Figure 9.**
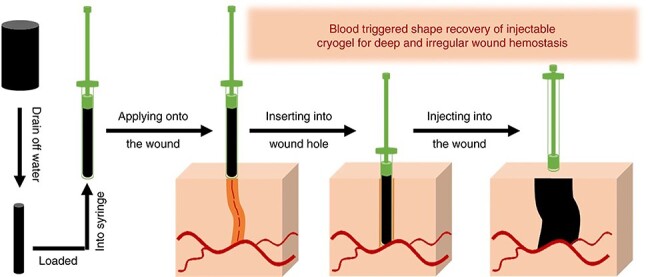
Recovery process of the OCSG/CNT cryogel in a deep and irregularly shaped wound [3] (open access). *QCSG* quaternized chitosan, *CNT* carbon nanotubes

Mussel foot proteins contain 3,4-dihydroxyphenylalanine, which can interact with substrates via strong covalent and noncovalent bonds; thus, the mussels have a strong capacity to adhere to wet surfaces [108, 109]. Liu *et al.* prepared a silica/polydopamine nanoparticle (PDA/SiNP) via lyophilization, and PDA/SiNP can be degraded 40% after 24 hours according to the in vitro degradation test [108]. Compared with the commercial Celox®, the hemostasis time of PDA/SiNP decreased by 150 seconds in an *in vitro* experiment. In a rat femoral artery and vein injuries models, the hemostasis time of PDA/SiNP was shorter than the control groups. In a rat liver model, the PDA/SiNP stopped bleeding in 86 seconds, which was faster than in the Celox group (about 102 seconds). In addition, the material displayed a long-term antibacterial ability against *E. coli* even after 208 hours. Therefore, PDA/SiNP has the potential to serve as a rapid hemostatic dressing. Based on the adhesive mechanisms of mussels and the chitosan-based adhesives, chitosan-graft-polypeptides were polymerized by different initiators. The copolymers displayed high lap-shear adhesion strength, 195.97 kPa on porcine skin, and high tensile adhesion strength, 642.7 kPa on bone. In a rat skin injury and bone fracture model, the copolymer exhibited good hemostatic efficacy and shortened the healing period (1 day on skin wounds and 20 days on bone fracture) compared with the control group (14 days on skin wounds and 60 days on bone fracture) [110].

Gecko feet have thousands of setae (fibril arrays) that can increase the adhesive force between gecko feet and various surfaces; therefore, gecko-like morphologies have been studied and used for developing hemostatic materials [111]. Mahdavi *et al.* modified the surface of poly(glycerol-co-sebacate acrylate) (PGSA) to imitate the morphology of gecko feet [112]. Gecko-based PGSA has been coated with a layer of oxidized dextran to promote tissue adhesion. The adhesive ability of this substance improved in an *in vitro* pig intestine tissue model and an *in vivo* mice abdomen subfascial tissue model relative to the unpatterned PGSA polymer. Therefore, the gecko-based PGSA adhesives have great potential to serve as a hemostatic material to seal wounds and replace sutures/staples.

**Superelasticity** Superelasticity is used to describe an extraordinary capacity of materials in shape transformation [113]. Superelastic materials can rapidly recover under a high compression (>80%) and withstand a load of more than 50,000 times its own weight; the elastic recovery of superelastic polymers is about 90% [114, 115]. Common superelastic materials include polymeric C_60_ [115], semicrystalline polymers [116], carbon nanofibers [117, 118] and thermostable nanofibrous aerogels [119]. Superelastic materials have been used in aerospace, soft robots and supercapacitors [120].

**Figure 10.**
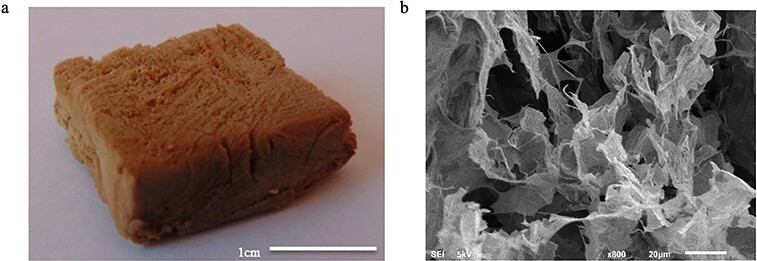
Picture **(a)** and scanning electron microscopy image **(b)** of the graphene oxide-poly(vinyl alcohol) aerogels [139] (Copyright 2018 by American Chemical Society, Washington, USA)

In battlefields, the limbs and joints of soldiers are the body parts most likely to receive penetrating and deep traumatic injuries [121, 122] that are difficult to repair or heal in a short time and may cause disability or death [123]. To deal with such trauma, an injectable hemostatic material with superelastic properties may match the shapes of uncompressed wounds and promote wound healing. Zhao *et al.* announced injectable antimicrobial conductive cryogels composed of carbon nanotubes (CNT) and quaternized chitosan (QCSG) [3]. The cryogel can rapidly recover its original shape upon contact with water (less than 1 second) and blood (Figure 9). The materials also have antimicrobial abilities, with 92%, 96% and 95% inhibition rates for *S. aureus*, *E. coli* and *Pseudomonas aeruginosa* (*P. aeruginosa*), respectively. *In vitro* blood clotting tests demonstrated that incorporating carbon nanotubes into QCSG can strengthen the blood clotting capacity and shorten the blood clotting index. *In vivo* hemostatic experiments in the mouse liver and tail amputation models and the rabbit liver volume injury model indicated that QCSG/CNT4 (cryogels with 4 mg/ml CNT) has a better hemostatic ability compared with Tegaderm™ film, such as quick hemostasis, lower blood loss and smaller wound surface.

Fan *et al.* prepared an injectable antimicrobial aerogel composed of oxidized cellulose carboxyl nanofibers and chitosan [122]. Because of the interlaced structure between nanofibers and nanosheets, the aerogel has high compressive strength (maximum 75.4 kPa) and a fast shape recovery capacity (recovery to its original shape within 30 seconds). An *in vitro* hemostatic performance test indicated the aerogel has excellent absorption and adhesion abilities for red blood cells and platelets.

Hydrogels can also be designed as a superelastic hemostatic materials because of their high hemostatic performance and biocompatibility. A conductive self-healing hydrogel wound dressing was fabricated from chitosan-g-polyaniline (QCSP) and poly(ethylene glycol)-co-poly(glycerol sebacate) (PEGS-FA) [124]. The hydrogels have a self-healing ability and their gelation time is 86 seconds. QCSP3/PEG-FA1.5: the hydrogel contains QCSP 30 mg and PEG-FA 15 mg has comparable ionic conductivity to that of human skin and muscles. Hydrogel QCSP3/PEGS-FA1.5 can inhibit over 99% of *E. coli* and 100% of *S. aureus* within 2 hours. In a mouse liver model, relative to the control group (about 2025 mg of blood loss), the hydrogel effectively stopped bleeding and reduced blood loss (only 215 mg). In a mouse skin lesion model, the hydrogel could repair the wound in 10 days, while the Tegaderm™ film did not heal the wound even in 15 days. Therefore, the hydrogels can serve as an effective hemostatic dressing.

Shape memory polymers (SMPs) have a shape recovery ability and can also serve as effective hemostatic materials for uncompressed wounds. Jang *et al.* designed a biodegradable SMP foam that is synthesized from triethanolamine and hexamethylene diisocyanate [125]. The SMP foams have a low density (0.076 g cm^3^), high gel fraction (over 90%) and a thermo-responsive shape recovery ability (recover to its original shape in 37 degree water for 8 minutes). The degradation experiment showed that the ester-containing foams can be completely degraded at day 90. Thus, the biodegradable capacity can help patients to avoid secondary surgery. Due to their porous structure, the mechanical strength of SMP foams was increased. Biodegradable SMP foams with clinically relevant thermal properties and rapid expansion performance have exhibited promising potential as hemostatic materials.

**High porosity (aerogel)** Aerogels have attracted numerous attentions because of its outstanding properties, such as ultralow density, wide surface area, high mechanical properties, high porosity and so forth [126–128]. Various materials have been used to prepare the aerogels, including silica [129], polyurethane [130], cellulose [131] and carbon [132]. The most common method for fabricating aerogels is direct freezing. In the freezing process, the microstructure of aerogels can be tuned by controlling external conditions like temperature. External forces can influence the microstructural growth of aerogels. Transverse magnetic fields, electrical fields and ultrasonic waves can cause different microstructures, namely, lamellar walls and mineral bridges, lamellar walls with long alignment and alternating complex rings, respectively [133]. Studies have demonstrated that aerogels have a high water absorption rate, fast shape recovery ability and high compressive mechanical strength [122]. Therefore, aerogels have been broadly used in varied fields, such as energy applications [134], drug delivery systems [135], skeletal muscle regeneration [136] and 3D printing [137].

Due to their high porosity and broad surface area, aerogels can be used in the hemostatic process and may have a similar hemostatic mechanism to ORC, that is, absorbing water when in contact with blood, forming a barrier at the bleeding site and serving as a matrix for clot formation [138]. Mellado *et al.* reported a composite aerogel, consisting of graphene oxide (GO) and poly(vinyl alcohol) (PVA), as a delivery system (Figure 10) [139]. The aerogel incorporates an extract from *Pai’s* grape seed (SD) and *Pai’s* grape skin (SK), as the extract has abundant proanthocyanidins that have the potential to promote wound healing. The absorption capacity is about 60 times the dry weight for GO-PVA aerogels, 70 times for GO-PVA-SD aerogels and 73 times for GO-PVA-SK aerogels. *In vitro* coagulant experiments showed that the GO-based aerogels started to coagulate from the beginning and that the aerogels with incorporated proanthocyanidins can completely coagulate the blood after 240 seconds. In the control group, coagulation of the blood began at 60 seconds and the blood was not completely coagulated after 240 seconds. The aerogels released 20% of their extract in 3 hours to promote wound healing, suggesting that the GO-based aerogels are a promising hemostatic material and delivery system.

**Figure 11.**
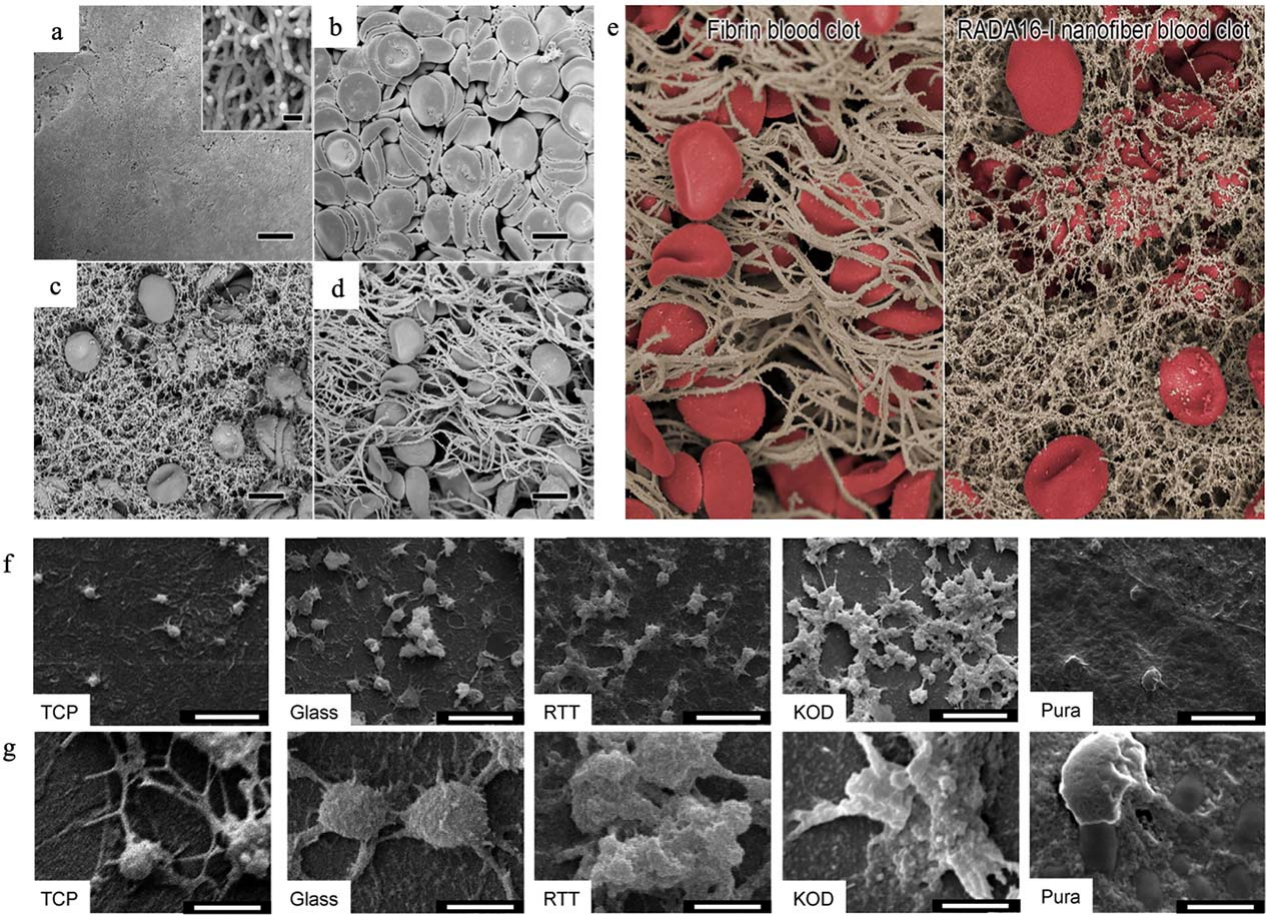
Scanning electron microscopy images of nanofibers with RADA16-I **(a)**, red blood cells and platelets in the anticoagulation whole blood **(b)**, RADA16-I nanofiber blood clot **(c)**, fibrin blood clot **(d)** and images of fibrin and RADA16-I nanofiber blood clots **(e)** [145]. **(f)** Low-magnification images of material surfaces adhering platelets indicating the platelet density; **(g)** high-magnification images showing platelet spreading and clumping to indicate the higher platelet activation [147] (Copyright 2014 by American Chemical Society, Washington, USA). *TCP* tissue culture polystyrene, *RTT* rat tail tendon, *KOD* collagen mimetic peptides, *Pura* Puramatrix, *RADA16-I* 16-residue peptide RADARADARADARADA

Another composite aerogel was prepared from dialdehyde nanocellulose fibers and collagen [140]. The study reported that the aerogels have desirable properties, such as a density of 0.02 g/cm^3^, a water absorption rate of 4000% and good biocompatibility. The average activity of L929 cells was 96.79% after culturing 5 days, demonstrating that the aerogels can promote cell proliferation. The aerogels have a higher porosity (95%) than the ideal porosity of hemostatic materials (at least 90%). Therefore, the nanocellulose fiber-based composite aerogels have a promising potential to act as hemostatic sponge materials and tissue engineering scaffolds.

**Polypeptide** Peptides are compounds composed of 2 to 50 amino acids and peptide bonds. A polypeptide contains 10 to 50 amino acids. Peptides have various applications, including medications, such as Acthrel®, Xerecept® [141] and antimicrobials [142].

Peptides can also be used in hemostatic materials. Although different hemostatic materials, such as chitosan, collagen, cellulose nanofibers and fibrin, have been developed and the commercial hemostatic products based on these materials can be found on the market (Table 1), their limitations also remain for clinical and emergency situations. Therefore, materials containing self-assembled peptides become an effective and alternative method. Self-assembled peptides are a kind of peptide that can organize each component spontaneously into a structure with certain sequences without external intervention [143]. Studies have demonstrated that self-assembling peptides can form nanofibers in solution to promote the coagulation process [144].

16-residue peptide RADARADARADARADA (RADA16-I) is a self-assembled peptide that can be used for hemostasis [145]. A layer-by-layer process was used to prepare a peptide-coated wound dressing. *In vitro* blood clotting experiments showed that RADA 16–1 and hemostatic materials (like gauze and gelatin sponge) coated with RADA 16–1 both can form nanofiber plug in rabbit red blood cells (Figure 11a, b, c, d, e). The porcine skin injury model indicated that peptide-coated gauze can stop bleeding within 2 minutes. Hemostatic bandages coated with RADA16–1 still release active nanofibers formed by peptides for hemostasis upon being exposed to harsh conditions (−80 to 60°C). Furthermore, Song *et al.* evaluated the hemostatic ability of RADA16–1 in a rat kidney model [146]. The results showed that, compared with Gelfoam (a commercial gelatin sponge), the blood loss in the RADA 16–1 group was reduced and less histological responses occurred.

Kumar *et al.* prepared self-assembled collagen mimetic peptides (KOD) to mimic the properties and structure of natural collagen for hemostasis [147]. The platelet adhesion experiment indicated that KOD adhere more platelets and form larger clots compared with control groups (Figure 11f, g). The soluble P-selectin secretion experiments demonstrated that KOD can active platelets. These properties are similar to those of natural collagen. Therefore, the self-assembled KOD have the potential to serve as wound dressings.

## Conclusions

Uncontrolled bleeding is a major cause of traumatic death. Hence, highly effective hemostats play an essential role in controlling hemorrhage and reducing the death rate in prehospital treatment. Commercial wound dressings, based on traditional hemostatic materials, including fibrin, collagen and zeolite, are available on the market. However, there are several disadvantages of these products, such as risk of infection, low tissue adhesion and secondary damage. High-performance hemostatic materials are, therefore, in demand to overcome these problems. Extensive research and development has been conducted in high-performance wound dressings to enhance hemostatic efficiency and promote wound healing. More work is needed to solve existing problems. For example, smart hemostatic materials are expected to monitor the status of wound healing and provide helpful information for doctors, advanced hemostatic materials are needed to stop internal bleeding (without open wounds) and they are also expected to have a longer shelf life and/or survive under extreme environments, such as high and low temperatures. Cost-effective hemostatic materials are also in great demand. Therefore, future studies of hemostatic materials may focus on the development of multifunctional and cost-effective hemostatic materials to meet different clinical requirements as described above.

## Abbreviations

GO: graphene oxide; HBP: hyperbranched polymer; hm-chitosan: hydrophobically modified chitosan; KOD: collagen mimetic peptides; NCFs: carbon nanofibers; ORC: oxidized regenerated cellulose; PDA/SiNP: silica/polydopamine nanoparticle; PDMAEMA: poly(N,N-dimethylamino-2-ethyl methacrylate); PGSA: poly(glycerol-co-sebacate acrylate); PHMB: polyhexamethylene biguanide; PVA: poly(vinyl alcohol); RADA16-I: 16-residue peptide RADARADARADARADA; SMP: shape memory polymer.
